# Surface mass balance analysis at Naradu Glacier, Western Himalaya, India

**DOI:** 10.1038/s41598-021-91348-3

**Published:** 2021-06-16

**Authors:** Rajesh Kumar, Shruti Singh, Atar Singh, Ramesh Kumar, Shaktiman Singh, Surjeet Singh Randhawa

**Affiliations:** 1 Department of Environmental Science, School of Earth Sciences, Central University of Rajasthan (CURAJ), N.H.8, Bandar Sindri, Ajmer, Rajasthan 305 817 India; 2 Department of Environmental Sciences, SBSR, Sharda University, Greater Noida, U.P. 201 306 India; 3 University of Aberdeen, King’s College, Aberdeen, AB24 3FX UK; 4 Himachal Pradesh Council for Science, Technology and Environment (HIMCOSTE), Vigyan Bhawan, Bemloe, Shimla, H.P. 171 001 India

**Keywords:** Climate change, Cryospheric science, Hydrology

## Abstract

In the present study, we analyze a field-based seven-year data series of surface mass-balance measurements collected during 2011/12 to 2017/18 on Naradu Glacier, western Himalaya, India. The average annual specific mass balance for the said period is  − 0.85 m w.e. with the maximum ablation of  − 1.15 m w.e. The analysis shows that the topographic features, south and southeast aspects and slopes between 7 to 24 degrees are the reasons behind the maximum ablation from a particular zone. The causes of surface mass balance variability have been analyzed through multiple linear regression analyses (MLRA) by taking temperature and precipitation as predictors. The MLRA demonstrates that 71% of the observed surface mass balance variance can be explained by temperature and precipitation. It clearly illustrates the importance of summer temperature, which alone explains 64% variance of surface mass balance. The seasonal analysis shows that most of the surface mass balance variability is described by summer temperature and winter precipitation as two predictor variables. Among monthly combinations, surface mass balance variance is best characterized by June temperature and September precipitation.

## Introduction

The importance of glaciers cannot be overlooked as they are key indicators of climate change along with providing fresh water to the downstream populations and maintaining the ecosystem. Worldwide, an increased global average temperature by 1.5 °C is causing enhanced melting of glaciers^1^. Rapid glacier mass loss may further cause changes in the landscape of mountains and Polar Regions that affect the global albedo and positively affect the global warming phenomenon. It also impacts local hazards, regional water cycles, and global sea-level rise^2–6^.

For more than a century, World Glacier Monitoring Service (WGMS) and its antecedent organizations collect and publish glacier fluctuation data obtained from its forty-one scientifically collaborating countries. The efforts have been made to gather long-term glacier observations, which would further give insight into climatic change processes, such as ice ages formation^7^. The critical work focus of WGMS is to collect standardized observations on changes in mass, volume, area, and length of glaciers with time. Also, it is deeply involved in providing statistical information about the distribution of perennial surface ice.

Glacier mass balance shows the most direct relationship between climate and glacier dynamics and consequently between climate and mountain hydrology^8,9^. It is a measurable unit and can be defined as the sum of glacial mass gain and loss. At present, mass balance studies are of great concern as they help monitor global climate change and explain rising sea levels^10–14^. Several glaciological parameters are being used to detail glacial response against climate change, but unfortunately, they are indirect and delayed^15^. In contrast, glacier mass balance is a natural and un-delayed process to detect climate change effects on the glaciers^16–21^. An extensive and continuous glacier mass balance study with more extended data series can help glacier results to be an indicator of climate variability^22^. The international research community views the study of glacier mass balance as necessary research nowadays because it is of an extensive belief that glaciers are losing mass^23–28^ due to global warming. In addition to this, understanding glaciers’ behavior against climate change is of enormous significance for assessing future water availability^29–32^. Glacier mass balance helps to understand the climate and improve our knowledge of the processes involved in Earth-atmosphere mass and energy fluxes. Mass balance studies are also valuable for estimating glaciers’ contribution to runoff and sea-level changes and making possible numerical models to analyze climate-glacier relationships^33^.

The Himalayan region comprises the largest glacier mass outside the polar areas, and this region is often referred to as the ‘water tower of Asia’. The role of Himalayan originated rivers in providing fresh water to the downstream population is very important, especially in the dry season^34,35^. Unfortunately, few mass balance studies have been done^36–38^ over different parts of the Himalayas. This shortcoming has been reported in the Himalayan region and the entire world^39^. The main objective of this study is to estimate the mass balance of Naradu Glacier, Western Himalaya, using the trendy glaciological method. The Glaciological mass balance of Naradu Glacier has been calculated for seven continuous years to understand its considerable contribution to the Baspa River and glacier sensitivity with changing climate.

### Study area

**Figure 1:**
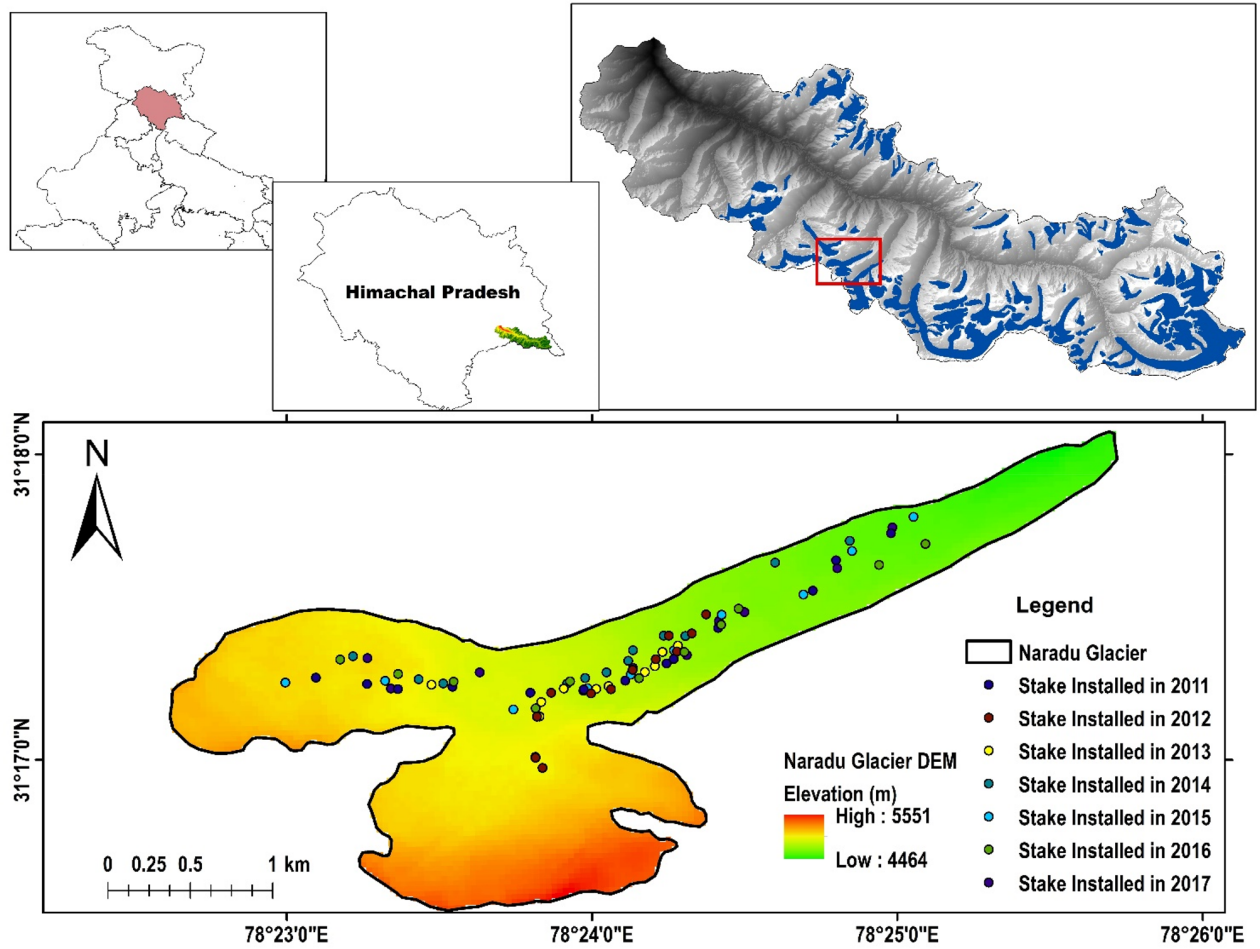
Location map of Naradu Glacier showing the network of stakes during the study periods. The map has been prepared using geographical information system (ArcGIS 10.1; version 10.1 and authorization number: EFL691568009-1010). (courtesy- India map shapefile at http://www.diva-gis.org/gdata; Digital Elevation Model (DEM) download from NASA Earth Data at https://search.earthdata.nasa.gov/search/; Naradu glacier shapefile digitized manually on Landsat 8 image acquired from USGS https://earthexplorer.usgs.gov/ dated 19 September 2019).

Naradu Glacier is among 89 glaciers of Baspa basin, western Himalaya^40^ and contributes its water to Baspa River. Baspa River joins Satluj River on its left bank near Karchham at an elevation of about 1770 m above sea level (m a.s.l.). Naradu Garang is a 3rd order stream of Sutluj and joins Baspa River on its left bank opposite Chitkul village at an elevation of about 3450 m a.s.l. The glacier ranges between 78° 25′ 06.17″ to 78° 25′ 34.07″ E and 31° 17′ 27.1″ to 31° 18′ 18.9″ N and covers an area of 3.8 km^2^^40^. It is a southwest-northeast facing glacier and falls in the SOI toposheet No. 53I/07. Naradu Glacier is highly debris (thin to thick cover) covered, and debris extends to 37.92% of the total glacier area. The location map of Naradu Glacier with a network of installed stakes during the study period is shown in Fig. 1 (prepared using geographical information system (ArcGIS 10.1; version 10.1 and authorization number: EFL691568009-1010).

### Climate dynamics of the valley

The Himalayan region’s hydrological cycle mainly depends on two circulation systems, Indian Summer Monsoon (ISM) and Western Disturbance (WD)^41–47^. Glaciers of western Himalaya have accumulated through WD mainly in January and February, while the eastern and central Himalayas' glaciers are accumulated mainly through the summer monsoon^48^. Western disturbance is the non-monsoonal precipitation driven by westerly wind directions, which brings sudden winter snow. The moisture of the western disturbance originates over the Mediterranean Sea^49^. In the winter months, western disturbances reach to lowest latitudes. In their way, they cross the north and central parts of India in a phased manner from west to east, disturbing usual features of the circulation pattern^50^. This causes snowfall in higher elevations of NW India and winter rainfall in plains of northern and central India. Baspa Basin falls in the western Himalayan Range and hence receives its precipitation during winter months due to westerly disturbances. The study region receives nearly 70% of annual precipitation as snowfall in winter and spring, and only 30% as rainfall near the glacier termini and as dry snow in higher-up regions^37^. The temperature analysis shows that the glacier’s monthly temperature ranges from  − 12.20 to 6.76 °C (Fig. S1a) between 2011–12 to 2017–18. The temperature trend analysis during 1979–80 to 2012–13 showed an increase of 0.9 °C in mean air temperature, whereas precipitation shows a decrease of 14.38 cm^40^.

### Data description and glaciological mass balance methodology

The most precise method for mass balance measurement is the glaciological method that utilizes the observations of differential exposure of installed stakes in the ablation zone to estimate the melting and digging pits to measure the accumulation. For our measurements, field visits were made during the last week of September to the first week of October during the study period. About 4 to 6 bamboo stakes (each 1.5 m long) were installed using a portable steam drill^51^ at different altitudes of the glacier in the ablation zone to measure the mass loss. The stakes’ differential exposure every year gave the annual vertical thinning of the glacier mass at that location. The multiplied annual exposure with the density of ice gives the specific mass balance at that glacier location. Density for ice is assumed constant at 900 kg m^−3^.

The variation between the beginning and the end of a hydrologic year represents the mass balance change for that year^52–55^. For the ablation measurement of Naradu Glacier, a network of stakes has been installed at different altitudinal zones (covering a range of 50 to 100 m). The average ablation in each zone is computed by multiplying the altitude band's area with the melting observed at the representative stakes.

For net yearly ablation measurement, the length of stakes above the glacier surface has been measured at two successive dates (t_1_ and t_2_). The depth of snow (D) over the ice surface was also measured. The difference between stake lengths buried in ice (L) and snow depths at t_1_ and t_2_ dates gives the specific ablation (ΔS) at that point. The exposure of stakes and snow depths were measured at each stake. The net ablation at a particular point is calculated by using the formula given below:

1 $$\Delta S = D_{i} \left[ {L\left( {t_{2 } } \right){-}L\left( {t_{1} } \right)} \right] + D_{s} \left[ {D\left( {t_{2 } } \right){-}D\left( {t_{1} } \right)} \right]$$

where $\Delta S$ = Specific ablation (m w.e.), $t_{1}$ = Year of initial measurement (cm), $t_{2}$ = Year of subsequent measurement (cm), $L =$ Length of stakes buried in ice (cm), $D =$ Depth of snow (cm), $D_{i}$ = Density of ice (g/cm^3^), $D_{s}$ = Density of snow (g/cm^3^).

Stakes above the glacier surface were measured every year from September 2012 to September 2018, with ice/snow density and the emergence difference giving the annual ablation at that point.

The ablation and accumulation values have been integrated over the glacier to calculate the mass balance. The overall mass balance, B_i_ is calculated according to:

2 $$B_{i} = S\sum b_{i} \left( {s_{i} } \right)$$

where b_i_ is the mass balance (m w.e.) of the altitudinal range *i* of area *s*_*i*_ (m^2^) and *S* is the total glacier area (km^2^). For each altitudinal range, *b*_*i*_ is obtained from the corresponding stake readings or net accumulation measurements.

## Meteorological data and MLRA

To better understand the causes of glacier surface mass balance (SMB) variability, multiple linear regression analysis (MLRA) is performed with temperature and precipitation series. For the analysis, monthly temperature and precipitation data have been downloaded from NASA GIOVANNI’s website (Fig. S1a). GIOVANNI is the acronym of Geospatial Interactive Online Visualization And aNalysis Infrastructure (Goddard Earth Sciences Data Information Services Center). It is an online (web) environment for the display and analysis of geophysical parameters. Data for both the parameters have been downloaded by selecting coordinates 31° 06′–31° 30′ N and 78° 12′–78° 37′ E with a grid size of 0.5° × 0.625° in the selected region. The GIOVANNI data has been carefully analyzed for homogeneity. ANOVA test has been used to check the inhomogeneity in temperature data. In contrast, due to the non-availability of real annual precipitation data, we cannot apply the same to precipitation data. ANOVA test has been used by considering the field observations (through AWS) of yearly temperature data for years 2012/13–2013/14 and 2015/16 to 2017/18. The test does not show any inhomogeneity as the calculated value is less than the table value of ‘F’ at the 5% level. Further, it has been assumed that GIOVANNI data for precipitation is homogeneous. The data shows a lower winter temperature (Fig. S1b). This may be the consequence of stronger winter inversion in the valley. An attempt has also been made to model the elevation against SMB. For the same, a best fit linear Eq. (3) has been estimated considering all stakes.

3 $${\text{SMB }} = \, 0.39 \, \left( { \pm 0.073} \right) \, *{\text{ Elevation }}{-} \, 2167 \, \left( { \pm 355} \right)$$

where SMB is the annual specific mass balance (m w.e.), elevation represents the stake elevation (m a.s.l.) and the uncertainties correspond to the 95% confidence level. Based on this simple linear fit approach, the average ELA for the period of 2011/12–2017/18 is obtained at 4914 m a.s.l., which is under-estimated compared to actual observations.

The stake’s elevation changes with time due to the melting of ice and glacial downward flow^56–58^. During seven years, the total elevation change is around 253 m for stake 1, 210 m for stake 2, and 183 m for stake 3 (kindly refer Statistical Analysis under Results and Discussion for Stake 1, 2 and 3 description). To analyze the effect of elevation change on surface mass balance, all stakes (i.e., 1, 2 and 3) are adjusted back to their initial elevation (i.e., 2011–12) by using Eq. (3). The observed and modeled surface mass balance (modeled surface mass balance is the surface mass balance obtained by putting various elevations in Eq. (3)) analysis shows a moderate correlation (R^2^ = 0.53). The modeled annual surface mass balance shows an average ablation of  − 2.48 m w.e. a^−1^, which does not significantly differ from the actual observed average ablation  − 2.34 m w.e. a^−1^.

## Results and discussion

### Accumulation and Ablation analysis

**Figure 2:**
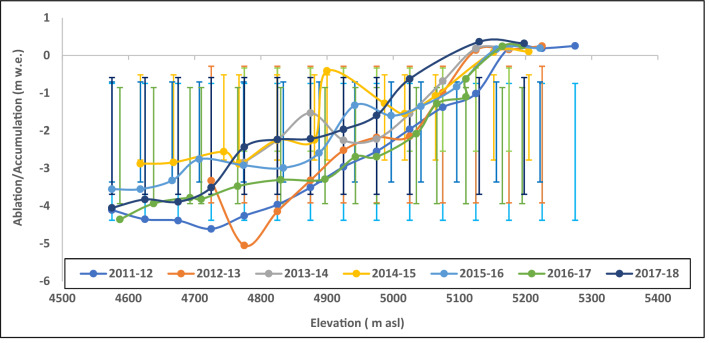
Specific accumulation/ablation with elevation during the ablation years 2011–12 to 2017–18. The vertical error bars indicate the standard deviation (± 1σ) of the accumulation/ablation.

The mass balance study on Naradu Glacier has started by Koul and Ganjoo previously under the DST-funded project during 2000/01–2002/03^37^. The second series of mass balance has been performed under the DST sponsored project No. SR/ DGH/HP-1/2009 dated 09.09.2010 for the year 2011/12–2013/14 followed by an extension of the activities for another four years (2014/15–2016/17) under project no. SB/DGH-92/2014 dated 19/02/2015. Also, one more year (2017/18) fieldwork has been performed to the Naradu Glacier to collect the data. The present study uses the most accurate glaciological data, and trendy methods to calculate the mass balance^59^ of Naradu Glacier for seven (2011–12 to 2017–18) years. In the entire study period, 84 annual surface mass balance measurements at different glacier locations have been performed. The specific ablation/accumulation with varying elevation in different years is shown in Fig. 2. All measurements show a negative mass balance. The variation in equilibrium line altitude (ELA) in various years and net mass balance is shown in Table 1.

**Table 1:** Mass balance results for the period 2011–12 to 2017–18.

Year	Net balance (10^5^ m^3^)	ELA (m a.s.l.)	Sp. Bal. (m w.e.)	Uncertainty (%)
2011/12	− 3.5	5209	− 1.09	2.6%
2012/13	− 3.7	5225	− 1.15	2.3%
2013/14	− 2.7	5196	− 0.86	1.3%
2014/15	− 2.5	5152	− 0.79	3.4%
2015/16	− 2.4	5135	− 0.77	1.6%
2016/17	− 2.0	5086	− 0.63	2.4%
2017/18	− 2.2	5127	− 0.69	2.1%
Average	− 2.71	5161	− 0.85	2.24%

**Figure 3:**
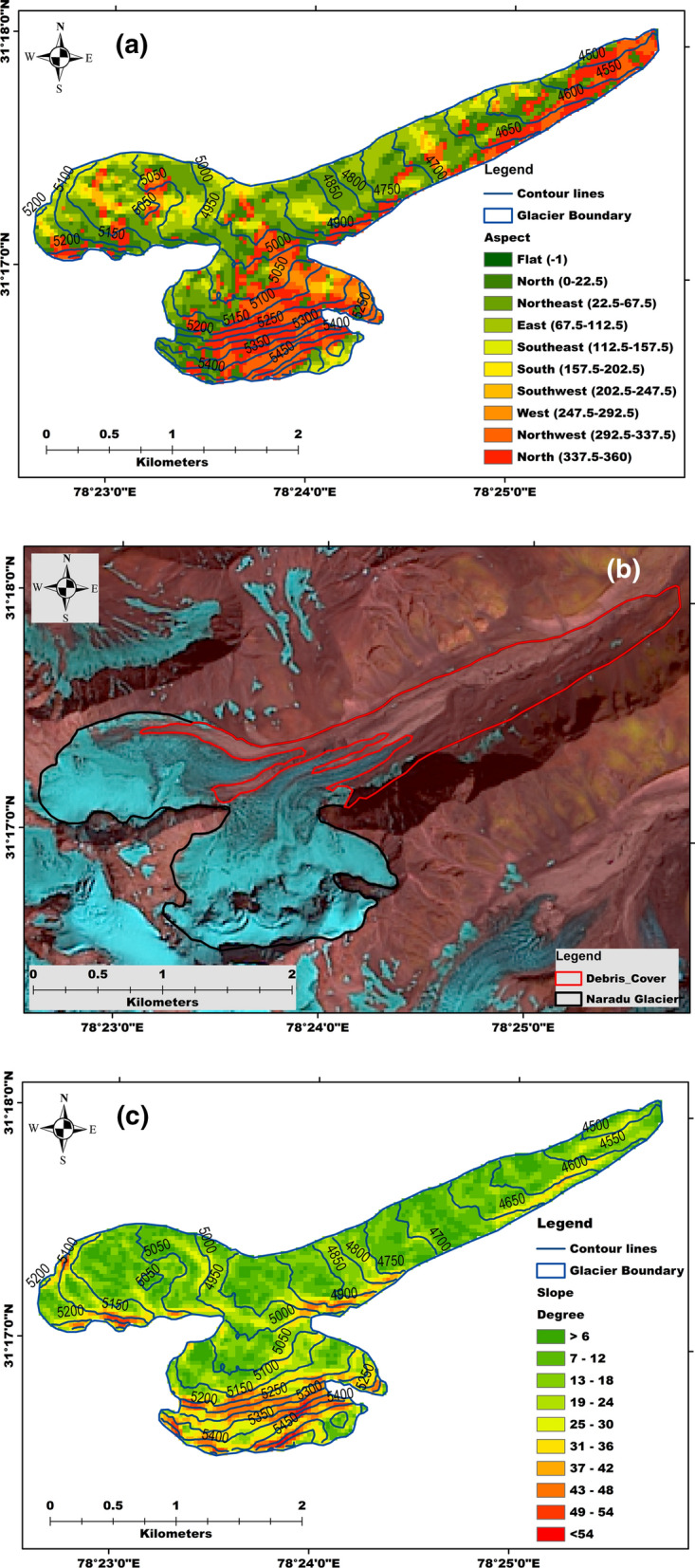
Naradu Glacier map showing (**a**) aspect, (**b**) debris-covered area, and (**c**) slope of different elevation zone. The map has been prepared using geographical information system (ArcGIS 10.1; version 10.1 and authorization number: EFL691568009-1010). (courtesy- Digital Elevation Model (DEM) download from NASA Earth Data at https://search.earthdata.nasa.gov/search/; Naradu glacier shapefile digitized manually on Landsat 8 image acquired from USGS https://earthexplorer.usgs.gov/ dated 19 September 2019).

The ablation estimation for the year 2011–12 is based on 13 stakes measurements, distributed between the elevation range of 4590 to 5136 m a.s.l. on the glacier’s central line. Ablation at a specific location was measured by observing the differential exposure of stakes yearly, preferable in the last week of September or 1st week of October, depending on the weather condition. Four pits between the elevation range of 5152 to 5289 m a.s.l. have been dug in the accumulation zone to obtain the annual specific accumulation. The subsequent years’ mass balance estimation is based on installing new stakes at new places and installing stakes to the earlier locations to have continuity in the data series. The observations are made to all stakes (new and old), where the old stake’s data provided the annual ablation of last year (2012–13). The new stake’s observation becomes the reference data for next year’s ablation measurements. The accumulation estimation for 2012–13 is based on four snow pits at the elevation range of 5132–5249 m a.s.l. Likewise, mass balance measurements for the years 2013–14, 2014–15, 2015–16, 2016–17, and 2017–18 have been made. During seven-year analysis, variation between lowest and highest melting is  − 0.1 m w.e. to  − 5.1 m w.e. The highest melting zone for four years (i.e., 2011–15) is at the elevation range of 4700–4800 m a.s.l. The topographic characteristics that play an important role in glacier melting are glacier hypsometry, slope and aspect^60^. Given this, we attempted to study all the possible topographic factors for Naradu Glacier, affecting the melting. We found that the south and southeast aspects, debris cover area, and the slopes between 7 to 24 degrees are the significant factors that would have made this zone the highest melting zone for four discussed years. The detailed map showing the aspect, debris, and slope of Naradu Glacier has been demonstrated through Fig. 3a–c (prepared using geographical information system (ArcGIS 10.1; version 10.1 and authorization number: EFL691568009-1010). During seven-year analysis, the year 2012–13 showed the highest ablation of  − 1.15 m w.e. supported by the detailed analysis of temperature indices showing comparatively high temperature during the same period. Along with temperature, net radiation, latent heat flux, and other topographical characteristics also played a significant role.

### Uncertainties of mass balance measurements

Worldwide, most of the mass balance calculations are done only for a few years, and large numbers of results are reported without uncertainty estimation^20^. A longer series of mass balance (more than 40 years) has been reported only for 33 glaciers^61^, and quality matters significantly in this kind of analysis. Various previous studies discussing errors in mass balance calculated by the glaciological method are in the record. Many authors estimated errors between ± 0.2 and ± 0.4 m w.e.^62–65^. Meier and others^66^ indicated errors between ± 0.1 and ± 0.34 m w.e. for mass balances determined by the glaciological method. Lliboutry^67^, calculated an error of ± 0.19 m w.e. for ablation measured with stakes, whereas ± 0.3 m w.e. of error was reported by Vallon and Leiva^68^. Gerbaux and others^69^ calculated the winter and summer balance and found an error of ± 0.10 m w.e. for ablation measured in ice and between  − 0.25 and + 0.4 m w.e. for ablation measured in firn. Error estimation in mass balance studies using the glaciological method is a very important issue. In this study, we have taken the utmost care in the measurements to reduce the possible errors. The error due to the movement of ice is negligible because of the low velocity of glacial ice. The errors in joining stakes and making a uniform surface at the bottom of the stakes have been carefully monitored and recorded.

Further to minimize the error in the average spatial result, many stake networks have been installed. In most mass balance studies, glacier area has been taken to be invariant, whereas it changes with time in actual practice and ultimately contributes to the overall error in the mass balance result^36,70^. To avoid this kind of errors, we have used the most recent area images to calculate the surface mass balance of every year. Further, the uncertainty related to the stake height determination, depth of snow in the ablation zone, and snow/ice density have been considered to calculate overall uncertainty in calculating the surface mass balance of Naradu Glacier. Uncertainty in the surface mass balance calculation of Naradu Glacier has been estimated using the equation suggested by Gantayat et al. (2014)^71^ and mentioned in Table 1.

### Statistical analysis

The study also aims to describe the observed surface mass balance (through MLRA) using temperature and precipitation as predictors. The additional predictors can be added, but it increases the fraction of surface mass balance variation, consequently reduces the degree of freedom. The p-value of the F-test should be as low as possible to justify the addition of predictors. The analysis is only based on continuous periods.

The multiple linear regression analyses has been conducted by taking 21 surface mass balance measurements. These 21 surface mass balances are based on three stake observations maintained by reinstalling to the following year’s location, in case it appeared that it would not survive till next year. The ablation zone of Naradu Glacier is a highly debris-covered area (refer to Fig. 3b) and may have a significant impact on the melting of ice/snow depending on its thickness^72^. MLRA does not include the surface mass balance measurements from the stakes that could not survive for the whole study period. The involvement of these kinds of measurements will surely raise the biases due to the gap in their data record^56^.

The specific mass balance measurements of three stakes are shown through Fig. S2 a and b. The annual ablation is more than 6 m w.e. for all the balance years except 2014–15. Modeled surface mass balance values show a significant increasing trend over seven years as the p-value of F-test is much lower than α = 0.01. The standard deviation in surface mass balance per stake per year varied between 0.002–0.17 m w.e. a^−1^ and does not show correlation with elevation (as R^2^ = 0.07) (Fig. S3).

For further analysis, the modeled surface mass balance measurements for each stake are converted to perturbations by taking seven years stakes’ mean. The surface mass balance perturbation is shown in Fig. S4. We found a perfect correlation between surface mass balance perturbation and elevation for all three stakes during analysis. Further, no link has been found between meteorological parameters (i.e., temperature and precipitation) and annual surface mass balance elevation gradient. The “no linkage” is a prerequisite condition for our analysis and is in line with many other related studies eg.,^73–76^.

To understand the relation between meteorological parameters and surface mass balance perturbation, the MLRA approach has been used by considering Eq. (4) ^57,77^. This correlation analysis requires the abandonment of the effect of measurement of different meteorological parameters in other units (here, the temperature in degree C and precipitation in mm w.e.). Hence, these parameters have been standardized by converting the data to z-score.

4 $$y = a_{1} x_{1} + a_{2} x_{2} + \cdots a_{n} x_{n} + b$$

where y = dependent/response variable and indicates surface mass balance perturbation in the present study. ‘a’ and ‘b’ are the regression coefficients, and x_1_, x_2_,…x_n_ are the independent/predictor variables. Here, $x_{1} {\text{and}} x_{2}$ is represented by the z-score of the meteorological parameters, i.e., temperature and precipitation. The monthly temperature and precipitation data show a weak correlation (R^2^ = 0.3), and this non-dependency is a common approach for MLRA performed on surface mass balance series e.g.,^56,78,79^. We have converted the meteorological data to a z-score. The regression coefficients ‘a_1_, a_2_…a_n_.’ show the climatic variability of meteorological parameters. Further, it has been assumed that the regression coefficients of both the parameters are uncorrelated and indicate the importance of both for surface mass balance. The intercept of the regression analysis (i.e., ‘b’) is equal to zero as it shows a value of y when all of the independent variables are equal to zero. The error degrees of freedom is the difference product of the total number of years (i.e., 7 in this analysis) and the number of independent variables used in the analysis (here temperature and precipitation). The outcome of MLRA is expressed in terms of R^2^ and p-value of the F-test. The factor R^2^ shows the variability of the response variable. The F-test performs a significant linear regression relationship between the response variable and the predictor variables. The p-value of the F-test is the probability of obtaining a linear correlation if the null hypothesis is true. The lower p-value at a higher significance level results in the rejection of the null hypothesis. We opted for a null hypothesis for analysis that there is no linear correlation between the response variable and the predictor variable.

Firstly, the annual average temperature (T_ann_) and total annual precipitation (P_ann_) have been used to explain the observed surface mass balance variation (MLRA with 5 error degrees of freedom). An MLRA shows that 71% of the variance of observed surface mass balance can be explained by these two predictors. The lower p-value of the F-test (0.07) describing the decisive significance of the model. The negative sign of T_ann_ shows a negative correlation between temperature and surface mass balance, and the positive sign of P_ann_ shows a positive correlation between precipitation and surface mass balance (refer to Fig. S5a and Table S1).

Secondly, the year is sub-divided into two categories, i.e., winter half-year (WHY) and summer half-year (SHY). The first category, i.e., WHY consists of fall (OND: October, November, December) and winter (JFM: January, February, March). The second category consists of spring (AMJ: April, May, June) and summer (JAS: July, August, September). The chosen monthly combination does not agree with meteorological seasons. They are selected according to the glaciological season so that the fall season (OND) should start just after field measurement.

The MLRA shows the importance of SHY temperature. This variable alone explains the 64% variance of observed surface mass balance (R^2^ = 64%; *p*-values F-test = 0.02). In the absence of this variable, no surface mass balance variance can be explained in MLRA with two predictor variables (For example, R^2^ = 58%; *p*-values F-test = 0.17) (Table S1). The summer temperature and winter precipitation account for 80% of the observed surface mass balance variance (with p-value of F-test 0.03), hence the null hypothesis, no linear correlation has been rejected. The larger absolute regression coefficient T_SHY_ (− 73.5) compared with P_WHY_ (+ 11.06) indicates a relatively higher importance of the SHY temperature (Fig. S5b).

Thirdly, the predictors split into seasonal components, i.e., spring (AMJ), summer (JAS), autumn (OND), and winter (JFM). This allows us to analyze 36 possible combinations for MLRAs using temperature and precipitation as a predictor variable. In the seasonal analysis, we found that with two predictor variables, most of the surface mass balance variability is described by summer temperature and winter precipitation (R^2^ = 82%; *p*-values F-test = 0.032) (refer to Table S1).

Depth analysis of all monthly combinations is also done, and the results show that the June temperature and September precipitation best describe the surface mass balance variance. This MLRA is statistically significant as it has much lower *p*-values F-test = 0.0031 and R^2^ = 94%. The individual monthly equation (refer to Fig. S5c and Table S1) indicates the dominance of temperature (regression coefficient of  − 50.51) compared with the September precipitation (regression coefficient of − 36.45).

### Temperature dominance

The analysis shows that the observed surface mass balance and temperature are strongly correlated. The same findings have been reported by Koul and Ganjoo^37^, in which they have assessed the impact of inter and intra annual meteorological parameters variation on Naradu Glacier mass balance. During the analysis, the authors have estimated that the melting of Naradu Glacier is positively proportional to the temperature, which is a function of solar radiation reaching on the glacier. Azam and others^80^ found that the turbulent heat flux has a significant impact on the surface mass balance of Chhota Shigri Glacier and is closely correlated with the temperature. The lack of such studies for nearby glaciers, which analyze the surface mass balance variability and its causes related to the meteorological parameters, restrict us to present more evidence in favor of the findings. The energy balance study of Naradu Glacier under the above-mentioned financial assistance has been done for five non-continuous years (2012–14 and 2015–18). We found that radiation mechanisms and sensible heat flux significantly drive the glacier’s specific energy balance in the analysis. This study finds that temperature explains a significant fraction of the observed surface mass balance because it is the representative index for solar radiation and sensible heat flux^48,81^.

The Naradu Glacier starts losing its mass from April and continues till September, and sometimes it extends till mid-October. In these months, along with the high temperature, the snow cover reduction also plays a vital role in glacier melting due to a decrease in albedo. The MLR analysis shows that the April to September months’ temperature and precipitation conditions significantly affect surface mass balance variability. Among all the monthly combinations, the variability is best described by June temperature and September precipitation. The precipitation during these months occurs as rain which further enhances the melting along with the high temperature.

### Naradu Glacier’s mass balance comparison with other glaciers of Indian Himalaya

The mass balance study on Naradu Glacier has been performed by using the most accurate glaciological method. Very few glaciological mass balance studies for a longer period have been reported in the Himalayan region^19,21,82^. The available field-based glacier mass balance data from Indian Himalayan regions are presented in Fig. S6. In Indian Himalaya, the Geological Survey of India (GSI) has started the detailed mass balance study using the glaciological method in 1974. The study was undertaken on Gara Glacier, Himachal Pradesh, to understand the glacial melt and its impact on local and regional hydrological systems. The Gara Glacier had been studied during 1974–75 to 1981–82^83,84^. The study showed a positive mass balance for the years 1974–75, 1975–76, and 1981–82 and the rest of the five years showed a negative mass balance. These positive mass balance results are dissimilar with most of the analyses done in the basin. The publication^83^ did not give any scientific reason behind this behavior of the glacier. Likewise, the Nehnar, Kashmir Himalaya glacier has been studied continuously for 8 years between 1975–76 to 1983–84. The study is one among many glaciers that has the longest glaciological mass balance record in the region. The scientific team involved in the study reported the negative mass balance for the entire study period, which ranges from  − 0.4 to  − 0.7 m w.e.^85^. The Shaune Garang Glacier has the longest study series (10 years or more) in the Baspa basin, showed a positive mass balance only for two years, and the rest eight years showed significant mass loss^86,87^. Later on, the reconstruction of mass balance on the same glacier has been done by Kumar and others^88^ for 2001–02 to 2007–08. In this reconstruction analysis, the authors found a negative mass balance for five years, whereas the glacier gained the mass in 2001–02 and 2004–05. On average, the results of Shaune Garang Glacier show more mass loss compared to Naradu Glacier. This high melting at Shaune Garang Glacier may be linked with the high temperature and lower precipitation conditions^89^. Another glacier with the longest study series outside the Baspa basin is the Chhota Shigri Glacier^90^ which is well-studied in many aspects. The glacier has been studied for mass balance, energy balance, and the reconstruction of the mass balance for over 43 years (1969–2012). The mass balance reconstruction for over 43 years was done to get the larger perspective of a glacier-climate relationship. The glacier reconstruction study shows that the ablation was more for most of the study years than the positive value. Likewise, the glacier’s mass balance using the glaciological method shows a negative mass balance for most of the study period. The marginal positive values were reported for the years 2004–05; 2008–09, and 2009–10^80^. Smaller duration mass balance studies have been reported from other glaciers like Rulung, Kolahoi II, Shishram, which showed the negative mass balance^19^.

Nine long-year analyses of the mass balance of Gor Garang Glacier, Baspa basin showed a negative mass balance for seven years and a minimal positive mass balance for two years^19,84^. The Dunagiri and Chorabari Glaciers of Uttarakhand Himalaya have been studied for six and more years and have been reported with negative mass balance^91–93^. The mass balance of Dokriyani Glacier for six years^92^ showed a negative trend. The reported reason was less winter precipitation, which causes longer period exposure of the glacier surface ice for melting. Less precipitation during the winter season leads to less input to the accumulation zone of the glacier. Hamta and Naradu Glacier of the western Himalayan region has been studied for 11 (2000–2009 and 2010–2012) and 3 (2000–2003) years, respectively. Both the glaciers showed a negative mass balance^37,94,95^.

### Other MLRA studies

Similar studies are limited in western Himalaya, which is extended throughout the Himalayan region^96^. The studies analyzing the effect of temperature and precipitation on surface mass balance variation found that surface mass balance is more sensitive to temperature rather than precipitation^56,97^. Still, the scenario may change depending on the spatial locations^98^, resulting in the change in the magnitude of various meteorological parameters. The same findings have been reported by Kayastha and others^99^. This study was done on Glacier AXOIO in the Nepalese Himalaya by taking three predictors: air temperature, precipitation, and relative humidity. The study results showed that mass balance is more sensitive to air temperature as apart from melting, it also controls the phase of precipitation (snow or rain). In 2017, Gaddam and others did the same study by taking four glaciers from the western Himalaya (three glaciers of the Baspa basin and one glacier from the Gara Khad basin)^89^. It has been reported that during the ablation season, the temperature perturbations were higher, whereas precipitation perturbations were higher during the accumulation season. The findings are same in our analysis, but there may be a difference in the magnitude of melting as the above study includes October month in the ablation period (in the present study, months from April to September defines the ablation season). Wang and others did a recent study at 45 glaciers of the Tianshan Mountains and Central Himalayan Mountains. They reported a linear increase in mass balance with the rise in perturbation of precipitation^100^. Our results are in agreement as the quantity and form of precipitation depend on temperature.

In 2015, Engelhardt and others^101^ analyzed four glaciers of Norway using the sensitivity formula given by^102^. In this analysis, Engelhardt found that at a higher temperature, surface mass balance sensitivity to temperature increases, whereas surface mass balance sensitivity to precipitation decreases. This shows that the sensitivity of surface mass balance also depends on the magnitude of temperature and precipitation; for example, higher temperature causes the reduction in the accumulation period and reduces the amount of precipitation as snow. Our continuous monthly period analysis shows a higher correlation compared with other studies^56^. This may happen because their analysis was based on many variables, i.e., May–June–July temperature and winter precipitation (here, we took June temperature and September precipitation). Apart from variation in the variables, the results also depend on the data quality (here, we took meteorological data from NASA GIOVANNI and field-based surface mass balance) and preprocessing before use. The validation of the satellite data with field data is a must for checking the homogeneity.

## Conclusions

This study uses the most accurate glaciological method to estimate the mass balance of Naradu Glacier of the Baspa basin for seven continuous years. The annual surface mass balance of Naradu Glacier for the period of 2011–12 to 2017–18 showed a negative trend with the maximum deficit of  − 1.15 m w.e. in 2012–13. The direct melting proportionality with the temperature makes this glacier witness to the higher sensitivity to temperature change. In Indian Himalaya, mass balance studies started back in 1974 and covered different glaciers for different periods. The studies reported so far confirm that almost all the glaciers under investigation have gone to a negative mass balance state except for a few with the marginal positive values (e.g., Gara Glacier, Shaune Garang Glacier, and Chhota Shigri Glacier). This indicates that the Baspa basin and the entire Indian Himalayas are experiencing a negative mass balance. Although in recent decades interest of the research community has increased to explore the glaciers of Indian Himalayan Region (IHR) yet the present study suggests that more attention should be given to glaciological mass balance studies as they are very few in numbers and hence the understanding of glaciers’ spatial and temporal variability is weak compared to the other world’s mountain glaciers. This study also describes the surface mass balance variation through MLRA by taking temperature and precipitation variables. The authors did not add other meteorological parameters (such as solar radiation, relative humidity, etc.) in the analysis as temperature and precipitation alone describe 71% of the observed surface mass balance variance. The research shows surface mass balance variation can be better characterized by summer temperature rather than precipitation. The summer temperature is an important variable explaining 64% variance of observed surface mass balance with *p*-values F-test = 0.02, which is quite satisfactory. The seasonal analysis with two predictor variables shows that most of the surface mass balance variability is described by summer temperature and winter precipitation (R^2^ = 82%; *p*-values F-test = 0.032). The monthly analysis indicates that high temperatures and low precipitation in June cause much of the snow to be melted out, exposing ice surfaces, resulting in lower albedo. Further, the type of precipitation (rain/snow) also influences the surface mass balance over the Naradu Glacier.

## Supplementary Information

Supplementary Information.
