# Supplementary material for: Surface mass balance analysis at Naradu Glacier, Western Himalaya, India
Source: Sci Rep. 2021 Jun 16;11:12710. doi: 10.1038/s41598-021-91348-3 (PMC8209027; doi:10.1038/s41598-021-91348-3)
Supplement: Supplementary file 1 — Supplementary Information. [file 41598_2021_91348_MOESM1_ESM.docx]

**Surface Mass Balance Analysis at Naradu Glacier, Western Himalaya, India**

**Rajesh Kumar^1, 2^, Shruti Singh^2*^, Atar Singh^2^, Ramesh Kumar^2^, Shaktiman Singh^3^ & Surjeet Singh Randhawa^4^**

*^1^Department of Environmental Science,* *School of Earth Sciences, Central University of Rajasthan (CURAJ), N.H-8, Bandar Sindri, Ajmer-305 817, Rajasthan, India*

*^2^Department of Environmental Sciences, SBSR, Sharda University, Greater Noida –201 306, U.P., India*

*^3^University of Aberdeen, King’s College, Aberdeen, AB24 3FX, United Kingdom*

*^4^Himachal Pradesh Council for Science, Technology and Environment (HIMCOSTE), Vigyan Bhawan, Bemloe, Shimla-171 001, H.P., India*

**^*^Corresponding Author:** shruti.singh.2229@gmail.com

**Figure S1. (a)** Monthly temperature and precipitation at Naradu basin; **(b)** Seasonal temperature and precipitation at Naradu basin.

**Figure S2: a)** surface mass balance against elevation in different years for three selected stakes (before projection to initial elevation); **b)** surface mass balance against elevation in different years for three selected stakes (after projection to initial elevation)

**Figure S3:** Standard Deviation of individual stake during 7 years 2011-12 to 2017-18

**Figure S4:** Surface mass balance perturbation for 3 selected stakes

**(a))**

**Figure S5:** Observed surface mass balance perturbation and modelled surface mass balance perturbation based on MLRA using two predictors **(a)** annual temperature and annual precipitation, **(b)** Summer half-year temperature and winter half-year temperature, **(c)** June temperature and September precipitation. The round cap solid blue line is the observed surface mass balance, and the round cap red dash line is the calculated surface mass balance signal resulting from the MLRA.

**Table S1:** Multiple regression analysis (MLRA) between z-score standardized meteorological variables and observed surface mass balance perturbations for three selected stakes covering the period 2011/12 – 2017/18

| **Period** | **Best-fit multilinear correlation** | **R^2^** | **p-value F-test** |
| --- | --- | --- | --- |
| **Annual** | SMB = -138*T_ann_ + 9.04*P_ann_ | 0.71 | 0.07 |
| **Half-year** | SMB = -59*T_SHY_ -7*P_SHY_ | 0.68 | 0.09 |
|  | SMB = -80*T_WHY_ + 9.04*P_WHY_ | 0.60 | 0.15 |
|  | SMB = -73.5*T_SHY_ + 11.06*P_WHY_ | 0.80 | 0.03 |
|  | SMB = 2*T_WHY_ -17*P_SHY_ | 0.56 | 0.19 |
| **Spring** | SMB = -51.97*T_spr_ + 0.49*P_Spr_ | 0.34 | 0.4 |
|  | SMB = -37.9*T_spr_ -19.06*P_Summ_ | 0.68 | 0.09 |
|  | SMB = -49.7*T_spr_ +19*P_Aut_ | 0.55 | 0.19 |
|  | SMB = -58.3*T_spr_ + 23.12*P_Win_ | 0.64 | 0.12 |
|  | SMB = -20.14*T_spr_ -14.19*P_SHY_ | 0.59 | 0.16 |
|  | SMB =-55.17*T_spr_ + 17.34*P_WHY_ | 0.76 | 0.05 |
| **Summer** | SMB = -103.1*T_summ_ – 3.57*P_Spr_ | 0.82 | 0.034 |
|  | SMB = -95.3*T_summ_ -1.07*P_Summ_ | 0.81 | 0.035 |
|  | SMB = -104.72*T_summ_ -4.16*P_Aut_ | 0.82 | 0.033 |
|  | SMB = -93.43*T_summ_ +4.45*P_Win_ | 0.82 | 0.032 |
|  | SMB = -106*T_summ_+2.04P_SHY_ | 0.81 | 0.035 |
|  | SMB =-96.1*T_summ_+0.77*P_WHY_ | 0.81 | 0.036 |
| **Autumn** | SMB = -15.42*T_Aut_ -15.28*P_Spr_ | 0.20 | 0.63 |
|  | SMB = -9.2*T_Aut_ -21.6*P_Summ_ | 0.51 | 0.23 |
|  | SMB = -0.90*T_Aut_+19.78P_Aut_ | 0.22 | 0.59 |
|  | SMB = -7.2*T_Aut_ +17.2*P_Win_ | 0.21 | 0.62 |
|  | SMB =1.3*T_Aut_ -17.4*P_SHY_ | 0.56 | 0.19 |
|  | SMB =28.9*T_Aut_ +22.18*P_WHY_ | 0.40 | 0.35 |
| **Winter** | SMB = -26.3*T_win_ -10.8*P_Spr_ | 0.24 | 0.56 |
|  | SMB = 16.4*T_win_ -20.1*P_Summ_ | 0.53 | 0.21 |
|  | SMB = -60.69*T_win_ +30.67*P_Aut_ | 0.66 | 0.11 |
|  | SMB = -44.9*T_win_ +22.2*P_Win_ | 0.47 | 0.27 |
|  | SMB = -0.90*T_win_ -17.1*P_SHY_ | 0.56 | 0.19 |
|  | SMB =-61.4*T_win_ +22.4*P_WHY_ | 0.28 | 0.03 |
| **SHY** | SMB = -107.6*T_SHY_ +12.3*P_Spr_ | 0.68 | 0.09 |
|  | SMB = -62.9*T_SHY_ -12.5*P_Summ_ | 0.75 | 0.05 |
|  | SMB = -77.1*T_SHY_ +10.4*P_Aut_ | 0.69 | 0.09 |
|  | SMB = -81.2*T_SHY_ +15.5*P_Win_ | 0.78 | 0.04 |
|  | SMB = -59.2*T_SHY_ – 7.3*P_SHY_ | 0.68 | 0.09 |
|  | SMB =-73.5*T_SHY_ +11.0*P_WHY_ | 0.80 | 0.03 |
| **WHY** | SMB = -135*T_WHY_ +11.24 *P_Spr_ | 0.45 | 0.29 |
|  | SMB = -54.8*T_WHY_ -15.86*P_Summ_ | 0.58 | 0.17 |
|  | SMB = -91.7*T_WHY_ +15.7*P_Aut_ | 0.56 | 0.19 |
|  | SMB = -88.61*T_WHY_ +11.75*P_Win_ | 0.49 | 0.25 |
|  | SMB = 2.43*T_WHY_ -17.6*P_SHY_ | 0.56 | 0.19 |
|  | SMB =-80.7*T_WHY_ +11.8*P_WHY_ | 0.60 | 0.15 |
| **Individual monthly combination** | SMB =-50.51*T_June_ - 36.45*P_Sep_ | 0.94 | 0.003 |

**Figure S6:** Annual specific glacier mass balance available in Indian Himalayan Region [98] and mass balance of the present study.
